# Microfluidization Preparation of Hybrid Graphene for Enhanced Wear Resistance of Coatings

**DOI:** 10.3390/polym17060824

**Published:** 2025-03-20

**Authors:** Qi Chen, Na Wang, Dhandapani Kuzhandaivel, Yingxian Chen, Lixin Wu, Longhui Zheng

**Affiliations:** 1 College of Chemistry and Materials Science, Fujian Normal University, Fuzhou 350007, China; 2 CAS Key Laboratory of Design and Assembly of Functional Nanostructures, Fujian Key Laboratory of Nanomaterials, Fujian Institute of Research on the Structure of Matter, Chinese Academy of Sciences, Fuzhou 350002, China; 3 Fujian College, University of Chinese Academy of Sciences, Fuzhou 350002, China

**Keywords:** graphene, microfluidization process, water-based coating, wear resistance

## Abstract

Wear resistance is the key factor that affects the long-term use of leather. Graphene has excellent wear resistance properties, but ensuring the effective dispersion of graphene in resin is crucial for determining the performance of the material. In this work, silica modified with polydopamine (SiO_2_@PDA) was used as an exfoliation agent. Using the microfluidization process and water as the medium, silica-graphene hybrid nanoparticles (SiO_2_@PDA-G) were prepared from expanded graphite. These nanoparticles were further compounded with waterborne polyurethane (WPU), and a superfine fiber-based fabric was used as the substrate to prepare composite coating. The results showed that the high shear force of the microfluidization process easily broke up the lamellar structure of graphite, resulting in few-layer graphene. Nano-silica was adsorbed on the surface of graphene, preventing re-aggregation between the graphene sheets. Compared to the WPU coating, the presence of SiO_2_@PDA-G improved the wear resistance and mechanical properties of the coating. The wear rate and the average friction coefficient of the composite coating decreased by 48% and 69%, respectively, and the tensile strength increased by 83%. Therefore, this study provides a new strategy for improving the dispersion of graphene in polymer materials and enhancing the abrasion resistance of the coatings.

## 1. Introduction

Abrasion can lead to substantial economic losses and a marked reduction in the quality of leather products, thereby affecting their durability, appearance, and overall value. To address this problem, various protective measures have been developed, with coating emerging as one of the most common and effective methods to safeguard leather surfaces from wear and tear.

In recent years, there has been a notable shift in the coating industry due to increasingly stringent environmental regulations. These regulations have led to a growing preference for water-based coatings over the traditional solvent-based alternatives. This transition is primarily driven by the need to reduce volatile organic compound (VOC) emissions and minimize the environmental impact of coating processes. Among water-based coatings, three main types have gained prominence in the leather industry: waterborne polyurethane (WPU) [1], waterborne epoxy resin [2] and waterborne acrylate [3]. Among them, WPU is composed of alternating monomer units connected by urethane bonds. The WPU represents a significant advancement in coating technology. Unlike conventional polyurethanes, which rely on organic solvents, WPU utilizes water as a dispersion medium. This fundamental difference in composition brings about several advantages that make WPU an attractive option for leather protection. One of the key benefits of WPU is their exceptional flexibility, which allows them to adapt to the natural movement and stretching of leather without cracking or peeling. This flexibility is crucial for maintaining the leather’s original feel and appearance, while providing a protective layer. Additionally, WPU exhibits excellent adhesion properties, ensuring that the coating remains firmly attached to the leather surface, even under challenging conditions.

In manufacturing, WPU has applications in protecting leather goods such as bags, shoes, and accessories. The furniture industry also benefits from WPU coatings, using them to enhance the durability and appearance of leather upholstery [4,5]. While WPU offers numerous advantages as an environmentally friendly coating material for leather protection, it is important to acknowledge that it still has some limitations. One of the primary challenges associated with WPU is its relatively poor water resistance compared to solvent-based alternatives. This limitation can be problematic in applications where leather may be exposed to moisture or high humidity levels. Another area where the WPU falls short is its low tensile strength [6]. This characteristic can potentially limit their effectiveness in protecting leather from severe abrasion or high-stress conditions. The inadequate wear resistance of WPU coatings is also a concern as it may result in the need for more frequent reapplication or maintenance of the protective layer.

Researchers have attempted a variety of methods to improve the wear resistance of coatings. The unique structure of hard and soft segments in polyurethane (PU) and the hydrogen bonding interactions between molecular chains make it possible to obtain various properties through molecular structure design. Zhu et al. [7] synthesized and used 6,6′-(1,4-butanediyl)-bis-(1,3,5-triazine-2,4-diamine) (BBTDA) to prepare crosslinked waterborne polyurethane (CWPU). By constructing a covalent bond crosslinking and hydrogen bond crosslinking network, the mechanical properties and wear resistance of the CWPU coating were enhanced. In addition, researchers have improved the wear resistance by preparing nanocomposite coating [8,9,10]. Wang et al. [11] used poly(2-butylaniline) (P2BA) as a dispersant. Through the non-covalent π-π interactions between P2BA and graphene nanosheets, they prepared P2BA-functionalized graphene (P2BA-G), achieving the stable dispersion of graphene in organic solvents. By compounding it with epoxy resin and carrying out a curing reaction under the action of an amine curing agent, the wear resistance and corrosion resistance of the coating were improved. Chen et al. [12] formed chemical bonds between functionalized graphene oxide (GO) and PU, thus, increasing the internal crosslinking degree of the composite coating. Moreover, by restricting the movement of polymer molecular chains through the anchoring and steric hindrance effects, they achieved high dispersion and high wear resistance of the composite material. Among the various approaches to address the improvement of coatings, graphene is the most interesting.

Graphene, due to its unique two-dimensional layered structure, high specific surface area, low friction coefficient, and excellent mechanical properties, can significantly enhance the mechanical and wear resistance properties of polymers, making it an ideal candidate for improving the wear resistance of polymer-based composites [13,14]. However, While various methods exist for fabricating graphene, such as liquid exfoliation [15,16], chemical oxidation/reduction [17,18,19,20], epitaxial growth [21,22], chemical vapor deposition (CVD) [23,24,25], and electrochemical exfoliation [26]. each approach is associated with limitations, such as high energy consumption, complex processing requirements, and environmental concerns. However, efficient way of producing graphene on a large scale is challenging. A promising novel technique known as microfluidization employs high pressure to generate forces that efficiently exfoliate graphite into graphene, thereby demonstrating the potential for large-scale production [27,28]. A primary obstacle in the effective utilization of graphene is its propensity for agglomeration owing to the inherently large specific surface area of graphene and the π-π conjugation between graphene sheets [29]. This aggregation can diminish the beneficial properties of graphene in composite materials.

To address these limitations, there is a need for improvements in eco-friendly coatings. In this work, a hybrid material based on graphene and silica was designed. To improve the surface properties of the silica, polydopamine-functionalized silica (SiO_2_@PDA) was formed through the in situ polymerization of dopamine on the surface of nano-silica particles. Using this as a functional additive and water as the medium, hybrid graphene was prepared by exfoliating expanded graphite via the microfluidization process. On this basis, the hybrid material was incorporated into WPU. Utilizing a superfine fiber base fabric as the substrate, wear-resistant graphene-infused superfine fiber artificial leather was prepared through the scratch coating method.

## 2. Experimental Section

### 2.1. Materials

Dopamine hydrochloride (DA) was purchased from Shanghai Aladdin Biochemical Technology Co., Ltd. (Shanghai, China); hydrophilic nano-silica (20 nm, 99 wt%) was purchased from Jiangsu XFNANO Materials Tech Co., Ltd. (Nanjing, China); Tris-HCl buffer solution was purchased from Beyotime Biotechnology Tech Co., Ltd. (Shanghai, China); expanded graphite powder (EGP) (1000 mesh) was purchased from Nanjing Greph Carbon Material Co., Ltd. (Nanjing, China); waterborne polyurethane (WPU) with a solid content of about 30% was purchased from Anhui Anda Huatai New Materials Co., Ltd. (Hefei, China); and the superfine fiber base fabric (Polyamide 6) was provided by Tianshou (Fujian) Superfine Fiber Technology Co., Ltd. (Longyan, China).

### 2.2. Preparation of Hybrid Graphene

Initially, 0.2 g of dopamine hydrochloride was solubilized in 100 mL of Tris-HCl (10 mm) buffer solution under constant agitation. Subsequently, 0.02 g of silica was incorporated and homogeneously dispersed. The resultant dopamine/silica suspension was then introduced into the inlet port of the microfluidization homogenizer. The suspension was subjected to high-pressure homogenization at 5000 psi and traversed through a nozzle and an emulsification chamber. This process was reiterated to yield an aqueous dopamine/silica dispersion. Subsequently, the pH of the dispersion was adjusted to 8.5, and the reaction was allowed to proceed under magnetic stirring at ambient temperature for 24 h, resulting in an aqueous dispersion of polydopamine-functionalized silica (SiO_2_@PDA). The subsequent phase involved the addition of expanded graphite powder (0.5 g) to the aforementioned aqueous solution, followed by iterative microfluidization treatment, comprising 30 cycles at 20,000 psi. This process culminated in the synthesis of a hybrid graphene, specifically, an aqueous dispersion of polydopamine functionalized silica-graphene, designated as SiO_2_@PDA-G.

### 2.3. Preparation of Hybrid Graphene/Polyurethane Composite Coating

**Figure 1:**
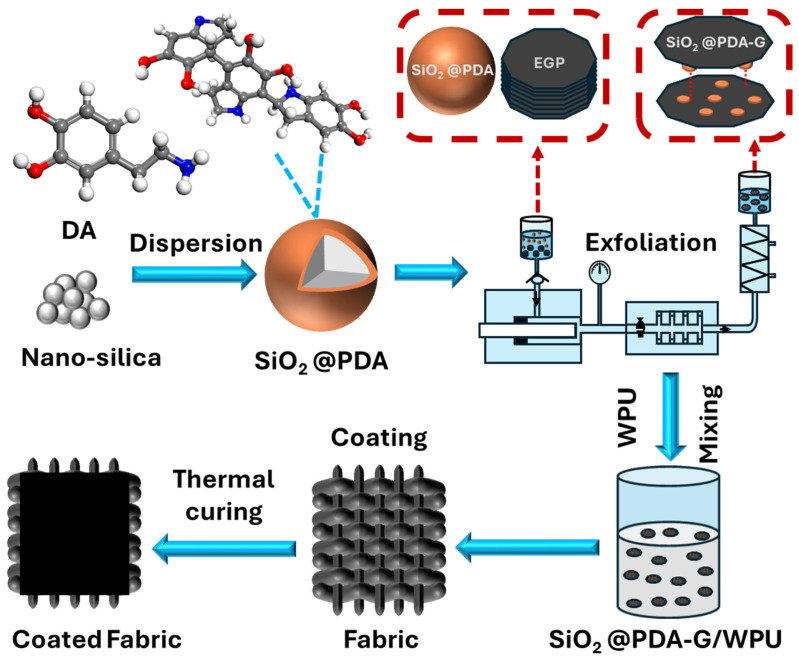
Schematic diagram of the preparation of composite coatings.

To prepare the composite coating, 10 g of SiO_2_@PDA-G aqueous dispersion was thoroughly mixed with 30 g of WPU and homogenized using a planetary vacuum homogenizer at 2000 r/min for 2 min. The resulting mixture was then uniformly coated onto a microfiber cloth using a 600 µm applicator. After each coating step, the sample was dried in an oven at 110 °C for 10 min. This coating and drying cycle was repeated 10 times to obtain the final SiO_2_@PDA-G/WPU composite coating. For comparison, a WPU coating was prepared following the same procedure, while a SiO_2_/EGP/WPU coating was fabricated by mechanically blending SiO_2_, EGP, and WPU. The detailed preparation process is illustrated in Figure 1.

### 2.4. Characterization

#### 2.4.1. Characterization of Hybrids

The morphological characteristics of the hybrid graphene were examined by Scanning Electron Microscopy (SEM, Sigma 300, ZEISS, Oberkochen, Germany) and Transmission Electron Microscopy (TEM, Talos F200S, FEI, Hillsboro, OR, USA). Elemental distribution analysis was conducted using energy-dispersive X-ray analysis (EDXA, Hillsboro, OR, USA) integrated with TEM. The thickness of the hybrid graphene sheets was quantified by Atomic Force Microscopy (AFM, Dimension ICON, Bruker, Billerica, MA, USA). The extent of surface defects in the hybrid graphene was evaluated through Raman spectroscopy (Raman, Labram HR Evolution, Horiba Jobin Yvon, Paris, France), utilizing a laser excitation wavelength of 532 nm. X-ray Diffraction (XRD, Ultima IV, Rigaku, Tokyo, Japan, using Cu Kɑ radiation, λ = 1.5406 Å, 40 kV and 40 mA) was used to elucidate the structure of the hybrids. The surface functional groups of the hybrid graphene were characterized using Fourier Transform Infrared Spectroscopy (FTIR, VERTEX70, Bruker Optics, Billerica, MA, USA) with a transmission module, covering a wavenumber range of 400–4000 cm^−1^. The samples were pre-prepared using potassium bromide pellet method and thoroughly dried prior to analysis. Thermogravimetric Analysis (TGA, HS-TGA-101, HeSon, Shanghai, China) was performed to assess the thermal stability of the samples. The analysis was performed under an oxygen atmosphere, with a temperature ranging from 30 to 1000 °C at a heating rate of 10 °C/min with a gas flow rate of 80 mL/min.

#### 2.4.2. Rheological Testing

The viscosity, storage modulus and loss modulus of the coating materials were tested using the polymer rheometer system (DHR-2, TA Instruments, New Castle, DE, USA).

#### 2.4.3. Characterization of Coating

The surface micromorphology of the coating was examined using scanning electron microscopy (EM-30+, COXEM Co., Ltd., Daejeon, Republic of Korea). The thermal stability of the coating was evaluated by thermogravimetric analysis (TGA HS-TGA-101, HeSon, Shanghai, China). The analysis was conducted under a nitrogen atmosphere with temperatures ranging from 30 to 700 °C at a heating rate of 10 °C/min with a gas flow rate of 80 mL/min.

#### 2.4.4. Wear Resistance Test of the Coating

The wear resistance of the composite coating was evaluated using a taber abrasion tester (CHK-5612, Guangdong AISRY Instrument Technology Co., Ltd., Dongguan, China). An H-22 type abrasive wheel was employed, and a load of 750 g was applied to conduct a 10,000-cycle abrasion experiment on the microfiber synthetic leather. An analytical balance was utilized to measure the mass of the coating before and after abrasion process. A pointer Shore hardness tester (LX-C-1, Wenzhou Weidu Electronics Co., Ltd., Wenzhou, China) was employed to assess the hardness of the coating, and the mean value was calculated after three measurements for each specimen. A friction tester (UMT-2, Silicon Valley, CA, USA) was used to determine the coefficient of friction of the coatings. An alumina ball was employed to abrade the coating surface for 10 min under a load of 5 N and friction rate of 200 mm/min.

#### 2.4.5. Tensile Property Testing

According to ASTM D638 standard [30], a universal testing machine (AG-X plus 1 KN, Shimadzu, Kyoto, Japan) equipped with a 1000 N load cell was used to test the mechanical properties of the samples, including tensile strength, Young’s modulus and elongation at break, at a strain rate of 10 mm·min⁻^1^. The coating mixture was injected into a dumbbell-shaped mold and cured at 110 °C to prepare tensile test specimens. The specimens have a total length of 115 ± 4.5 mm, with a gauge length of 65 ± 2.5 mm and a width of 6 ± 0.25 mm.

## 3. Results and Discussion

### 3.1. Preparation and Characterization of Hybrid Graphene

**Figure 2:**
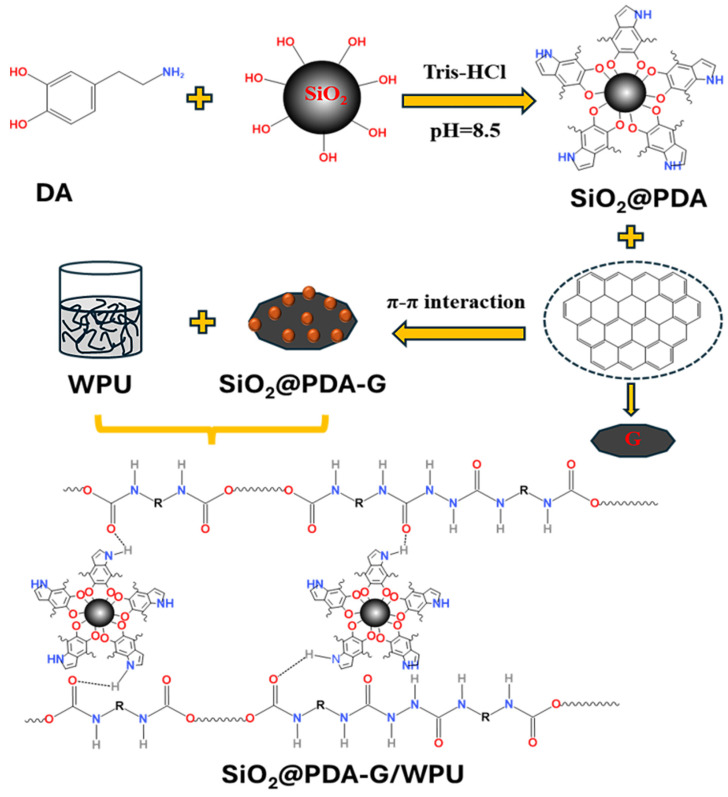
Fabrication principle of hybrid graphene/polyurethane composite coating.

The PDA can adhere to various material surfaces through covalent and non-covalent interactions [31], providing versatile reaction conditions for surface modification [32]. Herein, DA undergoes self-polymerization under weakly alkaline conditions to form PDA, whose catechol and amino groups bind to the hydroxyl groups on the SiO_2_ surface via hydrogen bonding, followed by dehydration condensation to form stable Si-O-C covalent bonds, thereby constructing a uniform PDA coating on the hydrophilic SiO_2_ surface and forming a core–shell structured SiO_2_@PDA. Furthermore, leveraging the π-π interactions between the aromatic rings in PDA and graphene, SiO_2_@PDA-G was prepared. Preparation principle of hybrid graphene was given in Figure 2.

**Figure 3:**
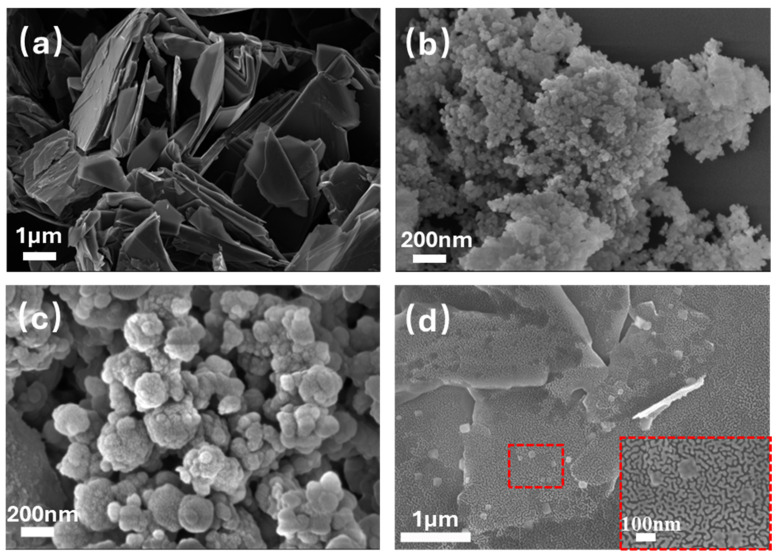
SEM images of EGP (**a**), SiO_2_ (**b**), SiO_2_@PDA (**c**), and SiO_2_@PDA-G (**d**).

**Figure 4:**
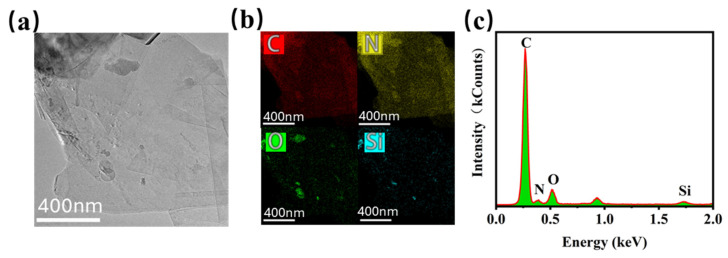
Characterization of SiO_2_@PDA-G using TEM image (**a**), Element mapping (**b**) and EDS (**c**).

Figure 3 illustrates the micromorphologies of EGP, SiO_2_, SiO_2_@PDA, and SiO_2_@PDA-G. Figure 3a depicts the surface morphology of EGP, revealing a lamellar structure with relatively thick and uniform layers. A comparison of Figure 3b,c demonstrates that PDA encapsulated the silica surface through self-polymerization. In contrast to Figure 3a, the SiO_2_@PDA-G layers exhibited a reduced thickness, with sporadic nanoparticles visible on the surface, indicating the adherence of SiO_2_@PDA to the graphene surface. The TEM micrograph (Figure 4a) reveals thin, semi-transparent graphene layers, which is a characteristic feature of few-layer graphene. This observation confirms the efficacy of microfluidization for exfoliating graphite to obtain few-layer graphene. Upon closer examination, it was found that a small quantity of nanoparticles adhered to the graphene surface. The elemental mapping and EDS results presented in Figure 4b,c indicate that these nanoparticles contain Si and N and were homogeneously distributed. Consequently, it can be deduced that these nanoparticles are SiO_2_@PDA. This inference is supported by the presence of numerous catechol structural units in PDA [28], which facilitated π-π interaction between PDA and graphene, resulting in the adsorption of SiO_2_@PDA onto the graphene surface and the formation of a hybrid structure.

**Figure 5:**
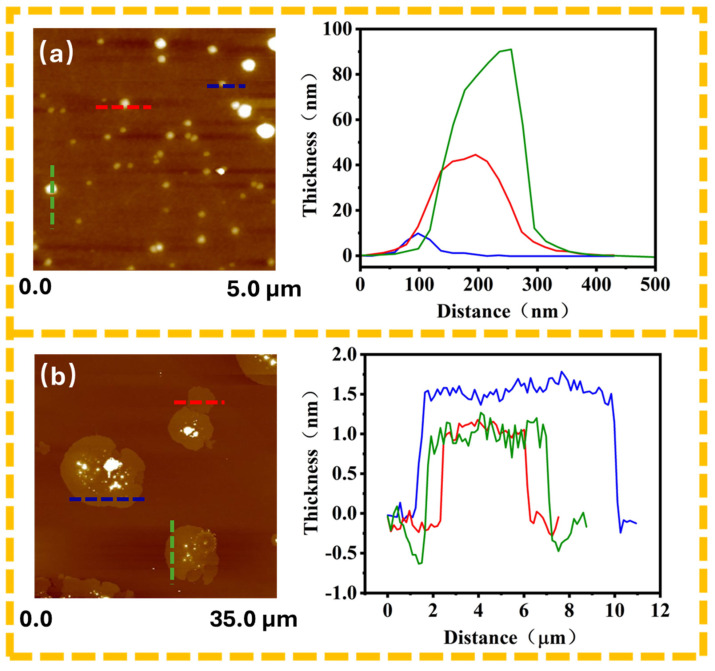
AFM images of (**a**) SiO_2_@PDA and (**b**) SiO_2_@PDA-G.

Atomic force microscopy (AFM) analysis provided valuable insights into the structural characteristics of the modified silica particles (SiO_2_@PDA) and graphene-coated silica particles (SiO_2_@PDA-G). The thickness distribution of the SiO_2_@PDA particles exhibited a wide range, from 10 to 90 nm, as illustrated in Figure 5a This variation in thickness suggests a nonuniform coating process or the presence of particle aggregates. In contrast, the graphene flakes displayed remarkable consistency in thickness, with measurements clustering around 1.2 nm, as shown in Figure 5b. This uniformity in graphene thickness is particularly noteworthy and indicates a well-controlled exfoliation process.

Further analysis of the graphene thickness provided crucial information regarding the number of layers present. Given that the theoretical thickness of a single graphene layer is 0.34 nm [33], the observed thickness of approximately 1.2 nm suggests that the graphene consists of 3 to 4 layers. This finding confirms the successful preparation of few-layer graphene through exfoliation of expanded graphite powder (EGP) using microfluidization. The achievement of few-layer graphene is significant, as it retains many of the desirable properties of single-layer graphene, while offering improved stability and ease of handling.

**Figure 6:**
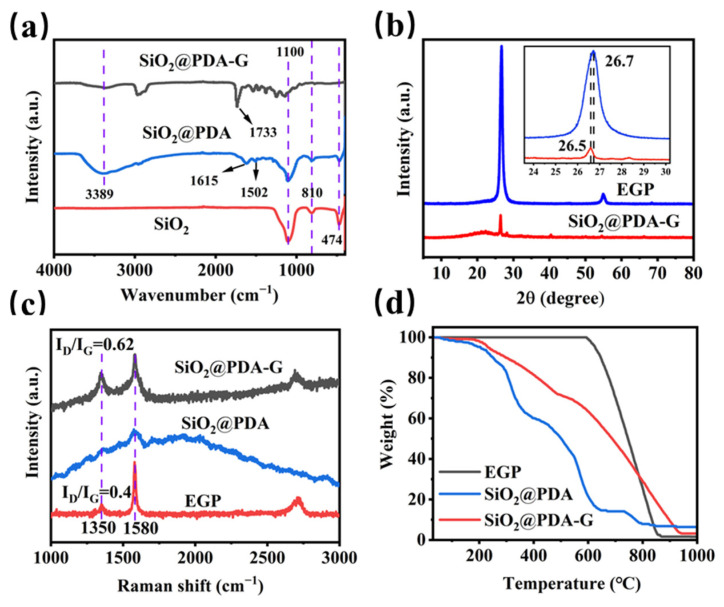
(**a**) FTIR; (**b**) XRD; (**c**) Raman; (**d**) TGA.

Figure 6a illustrates the FTIR spectra of SiO_2_, SiO_2_@PDA, and SiO_2_@PDA-G. The FTIR spectral analysis elucidated the molecular composition and interactions within the synthesized materials. The spectrum of SiO_2_ exhibited characteristic absorption bands at 1100, 814, and 474 cm⁻^1^, corresponding to the Si-O-Si asymmetric stretching vibration, Si-O-Si symmetric stretching vibration, and Si-O bending vibration, respectively [34]. These spectral features corroborated the presence of silica cores in the nanocomposite structure. Upon functionalization with polydopamine (PDA), novel absorption bands emerged in the infrared spectrum, indicating the successful encapsulation of SiO_2_ with PDA. The bands at 1502 cm⁻^1^ and 1615 cm⁻^1^ are attributed to the stretching vibration of C=C and the scissoring vibration of N-H, respectively [35]. These spectral signatures are indicative of the polydopamine structure, confirming its presence on the silica surface. Moreover, The SiO_2_@PDA-G exhibits a characteristic peak at 1733 cm^−1^, which may be attributed to the π-π interaction between the quinone structure (C=O stretching vibration) of PDA and the π-electron cloud of graphene. This leads to a redistribution of the electron cloud density of the C=O bond, resulting in a red shift in the vibrational frequency. This phenomenon can also be further confirmed by the peak shift observed in the UV-vis spectra (Figure S1).

The X-ray diffraction (XRD) analysis of EGP and SiO_2_@PDA-G (Figure 6b) provided significant insights into their structural characteristics. EGP exhibited a prominent (002) plane crystallization peak at 26.7°, indicating a well-ordered graphitic structure. Using Bragg’s formula [26], the interlayer was calculated as approximately 0.334 nm. Following microfluidization exfoliation, the SiO_2_@PDA-G sample exhibits a slight shift in the peak position to 26.5°, corresponding to an increased interlayer spacing of 0.335 nm. This subtle expansion suggested the successful intercalation of SiO_2_@PDA between the graphene nanosheets, demonstrating the efficacy of PDA in modifying the material structure.

Raman spectroscopy provides further evidence of the structural changes in the materials. The spectra of EGP, SiO_2_@PDA, and SiO_2_@PDA-G (Figure 6c) were analyzed, focusing on the characteristic G and D peaks of graphene at 1580 cm⁻^1^ and 1350 cm⁻^1^, respectively. The ratio of these peaks (I_D_/I_G_) serves as an indicator of the defect density in the graphene structures [36]. After modification, the I_D_/I_G_ ratio increased from 0.4 to 0.62, signifying an increase in defects within the graphene layers. This increase can be attributed to two factors: the physical impact of the microfluidization treatment on the graphite interlayer spacing and the influence of the SiO_2_@PDA modifier. The insertion of the modifier between the graphite layers likely caused changes in the interlayer distances and alignment, disrupting ordered stacking, and consequently increasing the number of defects [37]. These findings collectively demonstrate the successful modification of the graphene structure through intercalation of SiO_2_@PDA and microfluidization exfoliation.

In Figure 6d, thermogravimetric analysis (TGA) of EGP, SiO_2_@PDA, and SiO_2_@PDA-G revealed distinct mass-loss profiles across the four thermal regimes. In the initial phase (100–200 °C), SiO_2_@PDA and SiO_2_@PDA-G exhibited negligible mass reductions of approximately 3%, which was attributed to the desorption of physiosorbed water molecules. Notably, SiO_2_@PDA-G demonstrated a 1% lower mass decrease than EGP, indicating enhanced hydrophilicity conferred by PDA modification. The second thermal regime (200–600 °C) was characterized by a substantial mass reduction, primarily resulting from the thermal degradation and dissociation of PDA from the sample surface [38]. The third phase (600–850 °C) involved the thermal decomposition of both the PDA and graphene. A salient observation was made in the final thermal regime, which occurred above 850 °C. Although the thermogravimetric profiles of EGP and SiO_2_@PDA reached a plateau around this temperature, SiO_2_@PDA-G exhibited a pronounced lag, attaining stability only after 950 °C. This delayed stabilization indicates an augmented thermal resistance of the SiO_2_@PDA-G hybrid, which can be attributed to π–π interactions between SiO_2_@PDA and graphene. This enhanced thermal stability of the hybrid material suggests its potential applications in high-temperature environments where thermal resilience is paramount.

### 3.2. Rheological Property

**Figure 7:**
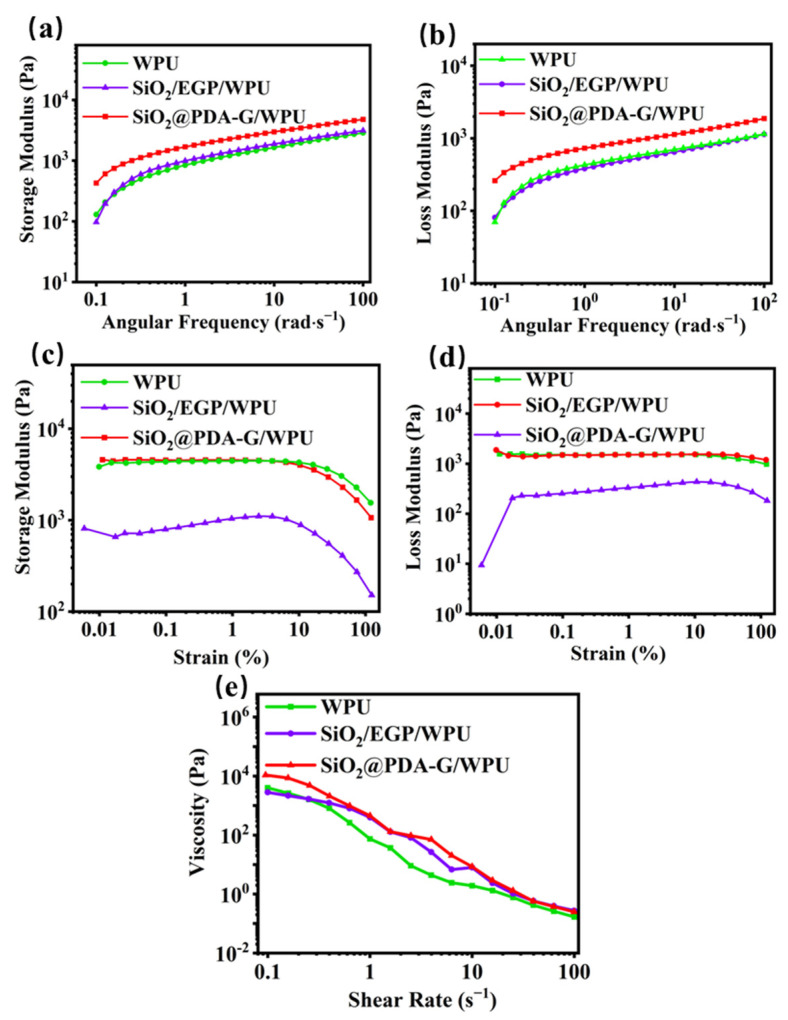
(**a**) Storage modulus-angular frequency curve, (**b**) loss modulus-angular frequency curve, (**c**) storage modulus-strain curve, (**d**) loss modulus-strain curve, (**e**) shear rate-viscosity curve of WPU, SiO_2_/EGP/WPU, and SiO_2_@PDA-G/WPU.

Rheological properties play a crucial role in influencing the preparation of coatings [39,40]. Figure 7a,b depict the relationships between the storage modulus (G′) and the loss modulus (G″) and the angular frequency within the linear viscoelastic region (strain = 0.1%). The results demonstrate that the G′ of the slurry exceeds G″ and predominates throughout the entire region (0.1–100 Rad/s), indicating that the slurry exhibits hydrogel-like behavior. This observation also substantiates the formation of a network structure facilitated by the abundant interfacial interactions between the WPU molecular chains and the modified graphene.

Figure 7c,d show the relationships between the storage modulus, loss modulus, and strain, respectively. The findings reveal that the coating slurry possesses an extensive linear viscoelastic region (approximately 10%), with entanglement network failure occurring only under high strain conditions (>10%). These characteristics suggest that the slurry exhibited a wide processing window and shear-thinning behavior. A comparative analysis of the storage modulus of the SiO_2_/EGP/WPU, SiO_2_@PDA-G/WPU, and WPU slurries with equivalent filler contents revealed that the storage modulus of the WPU and SiO_2_/EGP/WPU slurries were comparatively low. This observation further indicates that the interfacial interactions formed by the molecular chains in SiO_2_/EGP/WPU are less robust than those in the SiO_2_@PDA-G/WPU. Figure 7e illustrates the relationship between the slurry viscosity and the shear rate. The results demonstrated that the viscosities of all the tested slurries decreased as the shear rate increases. Notably, the SiO_2_@PDA-G/WPU slurry exhibited the highest viscosity at low shear rates, whereas the viscosity of the WPU remained low. This phenomenon may be attributed to the strong interfacial interaction between SiO_2_@PDA-G and the WPU molecular chains, which impedes their orientation and consequently results in a slower decrease in viscosity [41].

### 3.3. The Structure of Composite Coating

**Figure 8:**
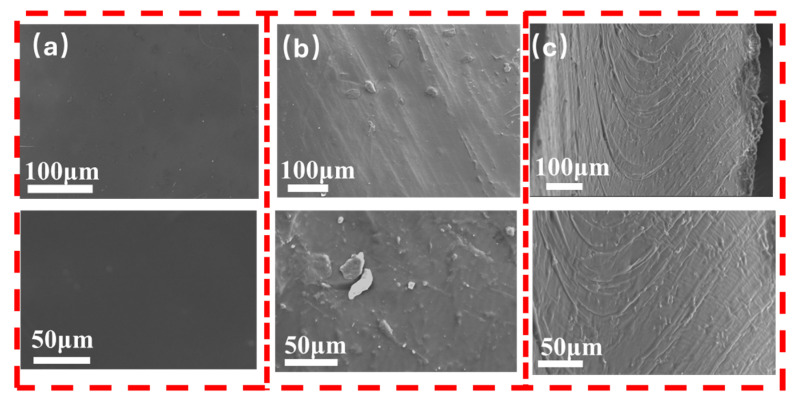
SEM images of the fractured surfaces of the coating ((**a**) WPU, (**b**) SiO_2_/EGP/WPU, (**c**) SiO_2_@PDA-G/WPU).

Cross-sectional SEM images of the coatings on microfiber artificial leather revealed distinct structural differences among the various coating compositions. The WPU coating (Figure 8a) exhibited a smooth and flat cross-section, indicating a uniform and homogeneous layer. In contrast, the SiO_2_/EGP/WPU coating displays a rough surface with visible expanded graphite flakes, suggesting a more complex and heterogeneous structure (Figure 8b). The SiO_2_@PDA-G/WPU composite coating (Figure 8c) exhibited the most intricate morphology, characterized by a wrinkled structure and nonlinear crack propagation patterns.

The unique features observed in the SiO_2_@PDA-G/WPU composite coating, particularly the wrinkled structure and ridge-and-groove-like stripes in the fracture cracks, indicate its superior mechanical properties. These characteristics suggest a strong interaction between the SiO_2_@PDA-G particles and WPU matrix, which can enhance the overall performance of the coating [42,43]. The wrinkled structure contributes to increased flexibility and stretchability while also serving as a mechanism for stress dissipation. When external forces are applied, the wrinkles act as buffers, distributing the stress more evenly throughout the material and preventing localized stress concentrations that could lead to damage [44]. This structural arrangement potentially improves the durability and resilience of the composite coating, making it more suitable for applications that require enhanced mechanical properties.

**Figure 9:**
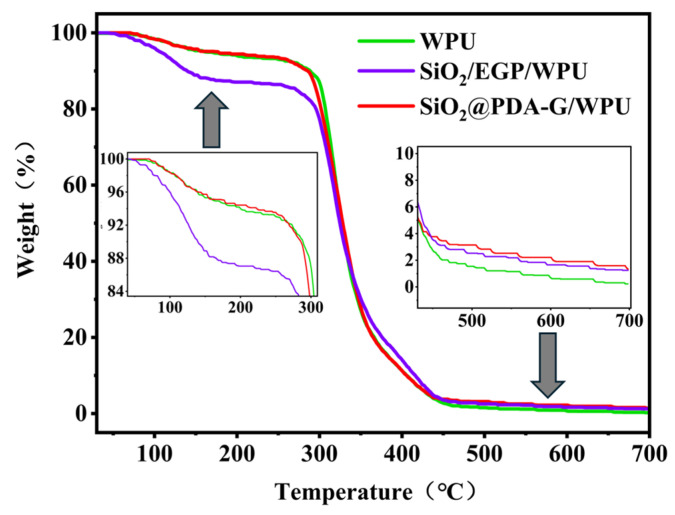
TGA curves of WPU, SiO_2_/EGP/WPU and SiO_2_@PDA-G/WPU.

Thermal stability of WPU and its composites was given in Figure 9. During the initial phase at 30–150 °C, all the samples exhibited evaporation of residual water. Notably, the SiO_2_/EGP/WPU composite demonstrated a more substantial mass loss within this range, which could be attributed to the excellent hydrophilicity of SiO_2_. The primary thermal decomposition of WPU and polydopamine (PDA) occurs within the 300–450 °C range. Interestingly, the SiO_2_@PDA-G/WPU composite material exhibited thermal stability comparable to that of pure WPU, suggesting that the incorporation of the hybrid did not compromise the overall thermal stability. This consistency in the decomposition rate indicates a favorable interfacial interaction between SiO_2_@PDA-G and WPU. The increase in interfacial interaction is confirmed by the water contact angle measurements for pure WPU, SiO_2_/EGP/WPU, and SiO_2_@PDA-G/WPU in Figure S2. The preservation of thermal stability in the composite is a significant finding, because it implies that the advantageous properties of the hybrid material can be integrated without compromising the thermal resistance of the original WPU coating.

### 3.4. Wear Resistance Performance

**Figure 10:**
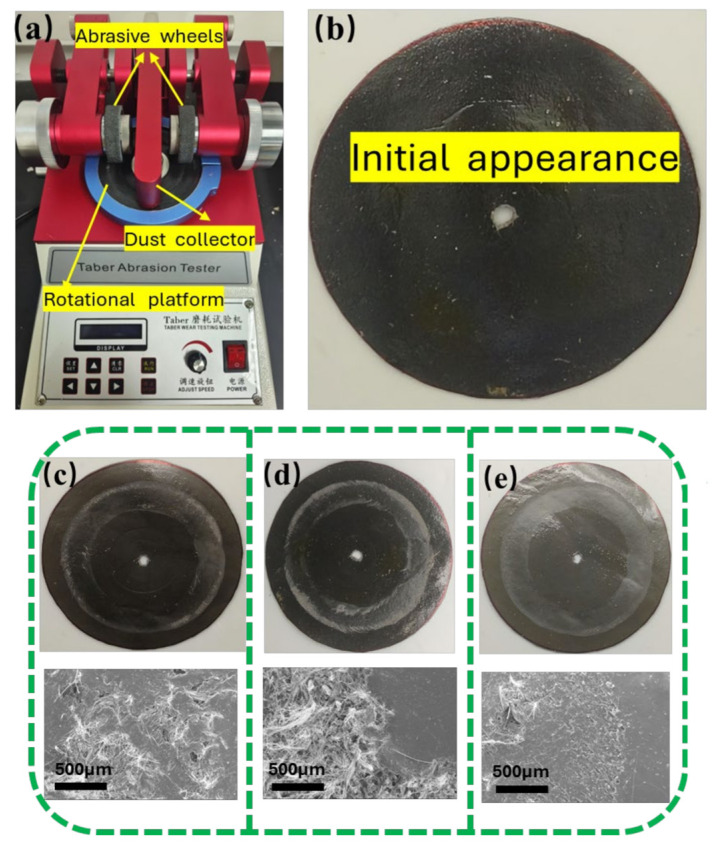
(**a**) Taber abrasion tester; (**b**) initial appearance of the sample; morphologies of WPU (**c**), SiO_2_/EGP/WPU (**d**), and SiO_2_@PDA-G/WPU (**e**).

**Figure 11:**
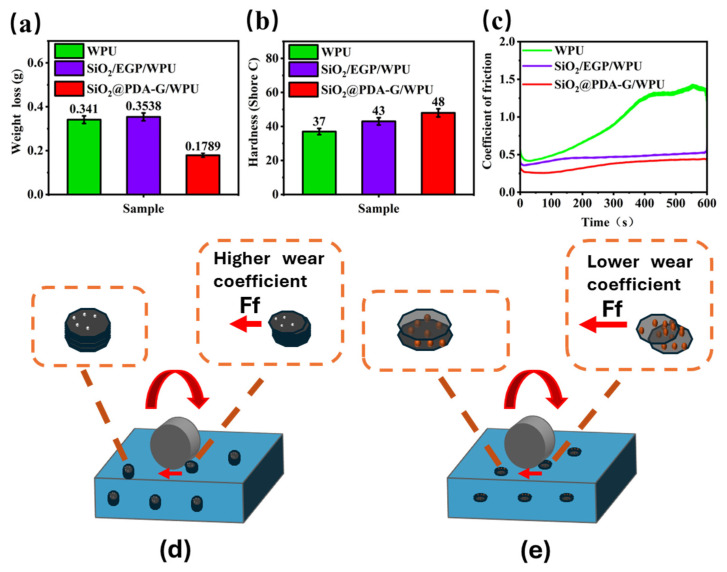
(**a**) Mass loss after abrasion measurement; (**b**) shore hardness (Shore C); (**c**) coefficient of friction; abrasion resistance mechanisms of SiO_2_/EGP/WPU (**d**) and SiO_2_@PDA-G/WPU (**e**).

Figure 10 shows the abrasion tester and the morphological diagrams of the coatings before and after the abrasion test. Figure 11a shows the mass loss of the samples after the abrasion test using the Taber abrasion tester. Among the evaluated specimens, SiO_2_/EGP/WPU exhibited the least favorable abrasion resistance, manifesting substantial fiber exposure after 10,000 abrasion cycles and a mean mass loss of 0.3538 ± 0.0027 g. Thus, the SiO_2_@PDA-G/WPU composite coating demonstrated superior performance, with a minimal relative mass loss of 0.1789 ± 0.0016 g following an equivalent number of cycles. This represents a 48% enhancement in wear resistance compared to that of the original material. Microscopic examination of the SiO_2_@PDA-G/WPU coating surface revealed only minor surface degradation and minimal exposure of the microfiber cloth fibers. Subsequent analysis utilizing Shore durometers corroborated the exceptional properties of the SiO_2_@PDA-G/WPU composite. Hardness assessments conducted on WPU, and its composite materials indicated that SiO_2_@PDA-G/WPU attained the highest hardness value of 48C. This superior hardness, in conjunction with the minimal mass loss and surface deterioration observed during abrasion testing, provides compelling evidence for the exceptional wear resistance of the SiO_2_@PDA-G/WPU composite coating. These findings suggest that the incorporation of SiO_2_@PDA-G particles into the WPU matrix significantly augments the durability and resistance of the material to mechanical wear, rendering it a promising candidate for applications requiring robust and long-lasting protective coatings.

The mechanism underlying the enhancement of the composite wear resistance by graphene encompasses two primary aspects. Firstly, it stems from graphene’s inherent properties, including its lamellar structure, exceptional mechanical characteristics and low surface friction coefficient [45,46,47,48]. Secondly, it is contingent on the aggregation state of graphene sheets within the polymer matrix, encompassing spatial dispersion uniformity and interfacial interactions.

The augmented wear resistance of waterborne polyurethane/graphene composites results from the synergistic effect of its intrinsic properties and optimized aggregation state. The chemically modified graphene surface exhibited a low friction coefficient, consequently imparting a relatively low friction coefficient to the composite material during the abrasion contact process (Figure 11c). Moreover, the unique two-dimensional lamellar structure of graphene provides an extensive specific surface area. Surface-modified graphene facilitates uniform dispersion within the matrix, mitigating agglomeration induced by π-π interactions. This optimization of the effective specific surface area, strengthens the interface with the polymer, thereby enhancing the capacity to transfer destructive stress, Consequently, it dissipates wear energy and suppresses wear intensity through mechanisms such as stress-induced crack tip blunting, deflection, bridging and crack-propagation inhibition [49,50,51] (Figure 11d, red arrows and circles). When externally induced wear force propagate cracks within the composite, thin-layer graphene attenuates the crack strength owing to its inherently robust mechanical properties. Concurrently, the favorable lubricity between the graphene sheets facilitates crack deflection. This process culminates in energy dissipation, reducing the destructive potential of cracks and enhancing the abrasion resistance.

Conversely, the incorporation of un-exfoliated expanded graphite with silica (Figure 11e) presents challenges. The relatively thick lamellae impede deflection, resulting in an increase in the friction coefficient. Consequently, more energy was expended in abrading the agglomerated graphite and free silica, thereby facilitating their wear. Pure waterborne polyurethane exhibited the highest friction coefficient. In the absence of reinforcing elements to suppress stress-induced cracks, all cracks propagate unimpeded within the matrix, leading to catastrophic damage [52].

### 3.5. Tensile Testing

**Figure 12:**
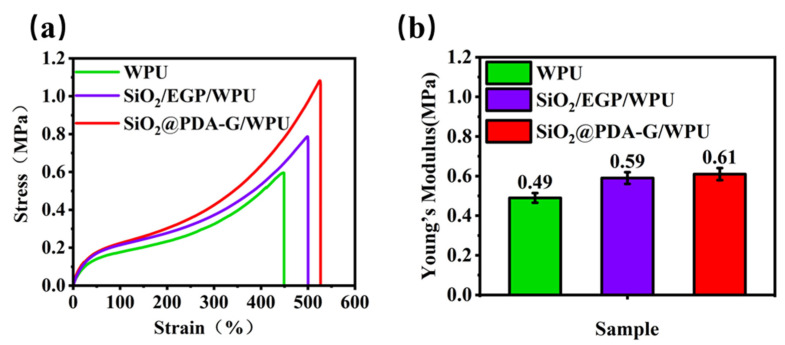
(**a**) Tensile stress–strain curves and (**b**) Young’s modulus of WPU, SiO_2_/EGP/WPU, and SiO_2_@PDA-G/WPU.

The filler loading, quality of filler dispersion, and interfacial interaction between the filler and WPU polymer matrix are critical parameters that significantly influence the ultimate mechanical properties of composite materials [53]. Figure 12 illustrates the mechanical properties of the WPU, SiO_2_/EGP/WPU, and SiO_2_@PDA-G/WPU. It is evident that the incorporation of SiO_2_ and EGP into WPU matrix through blending resulted in enhanced mechanical properties of the SiO_2_/EGP/WPU composite coating. Notably, SiO_2_@PDA-G/WPU exhibited superior tensile strength, elongation at break, and elastic modulus compared with the other compositions. The tensile strength increased by 83% relative to the original value, whereas the elongation at break improved by 18%. This enhancement can be attributed to the high adhesion of PDA and its strong interaction with the urethane and urea groups of the WPU hard segments, which augmented the interfacial interaction between SiO_2_@PDA-G and WPU. Furthermore, the graphene sheets contributed to the formation of a self-lubricating and high-strength continuous transfer film during the wear process because of their low coefficient of friction and excellent lubricating properties, thereby improving the wear resistance of WPU. Additionally, the synergistic effect of graphene and silica effectively impedes crack propagation and delays the fatigue wear.

A comparative analysis of the mechanical properties of SiO_2_/EGP/WPU and SiO_2_@PDA-G/WPU revealed that surface-modified graphene exhibited superior reinforcement effects compared to unfunctionalized graphite powder. This phenomenon can be ascribed to the presence of numerous reactive functional groups on the functionalized graphene surface, which enhances the interfacial interaction with the polymer matrix and mitigates phase separation, resulting in improved stress transfer efficiency. Moreover, the amino-group-modified graphene surfaces demonstrated enhanced hydrophilicity, facilitating better dispersion within the waterborne polyurethane matrix and effectively preventing the agglomeration caused by π-π interactions. In conclusion, the synergistic effects of enhanced interfacial interactions and improved dispersion performance significantly contributed to effective stress transfer, thereby augmenting the strength and modulus of the polymer matrix.

## 4. Conclusions

In conclusion, this study demonstrated the successful preparation of hybrid graphene using SiO_2_@PDA as an exfoliation agent and water as the medium by employing microfluidization technology. The resulting few-layered graphene modified with SiO_2_@PDA was effectively incorporated into a WPU matrix. The graphene-reinforced WPU composite coating exhibited significant improvements in mechanical and tribological properties compared to pure WPU, including a 48% reduction in wear rate, 69% decrease in the average coefficient of friction, and 83% increase in tensile strength. These findings highlight the potential of this graphene composite coating for applications in wear-resistant leather coatings, offering a promising solution for enhancing durability and performance in this field.

## Data Availability

The original contributions presented in this study are included in the article. Further inquiries can be directed to the corresponding author.

## Supplementary Materials

The following supporting information can be downloaded at: https://www.mdpi.com/article/10.3390/polym17060824/s1, Figure S1: UV-vis spectra of SiO_2_, SiO_2_@PDA and SiO_2_@PDA-G; Figure S2: Contact angle of WPU (a), SiO_2_/EGP/WPU (b) and SiO_2_@PDA-G/WPU (c).

## Abbreviations

The following abbreviations are used in this manuscript:

EGP	Expanded graphite powder
WPU	Waterborne polyurethane
PDA	Polydopamine
G	Graphene
