# Supplementary material for: Microfluidization Preparation of Hybrid Graphene for Enhanced Wear Resistance of Coatings
Source: Polymers (Basel). 2025 Mar 20;17(6):824. doi: 10.3390/polym17060824 (PMC11944417; doi:10.3390/polym17060824)
Supplement: Supplementary file 1 [file polymers-17-00824-s001.zip › polymers-3494993-supplementary.pdf]

Supplementary Information

# Microfluidization preparation of hybrid graphene for enhanced wear resistance of coatings

Qi Chen [a, b, c], Na Wang [b, c], Dhandapani Kuzhandaivel [b, c], Yingxian Chen [b], Lixin Wu[*b] and Longhui Zheng[*b]

[a] *College of Chemistry and Materials Science, Fujian Normal University, Fuzhou 350007, China.*

[b] *CAS Key Laboratory of Design and Assembly of Functional Nanostructures, Fujian Key Laboratory of Nanomaterials, Fujian Institute of Research on the Structure of Matter, Chinese Academy of Sciences, Fuzhou 350002, China.*

[c] *Fujian College, University of Chinese Academy of Sciences, Fuzhou 350002, China.*

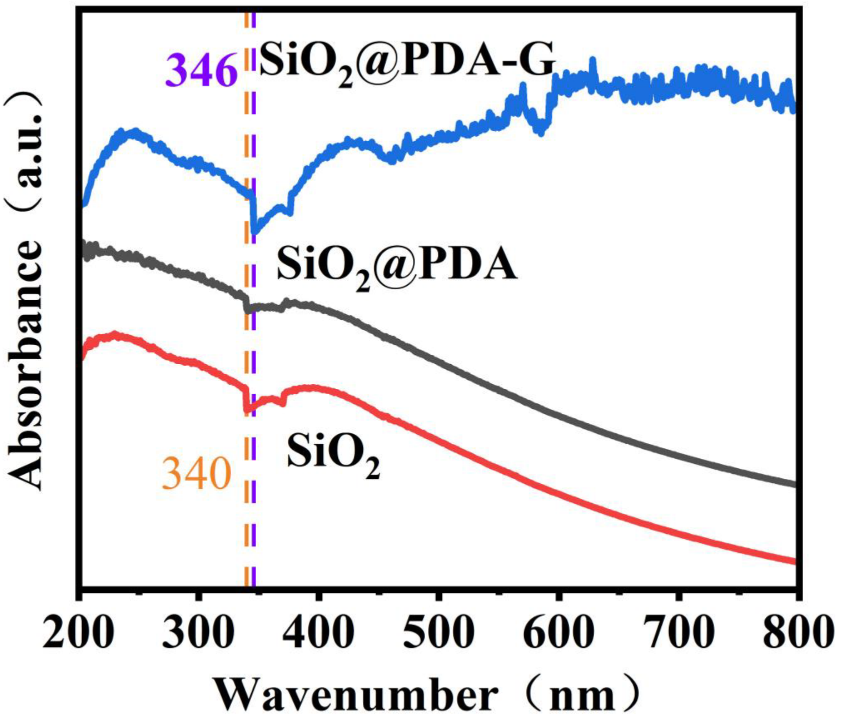


Figure S1. UV-vis spectra of $SiO_2$, $SiO_2$@PDA and $SiO_2$@PDA-G.

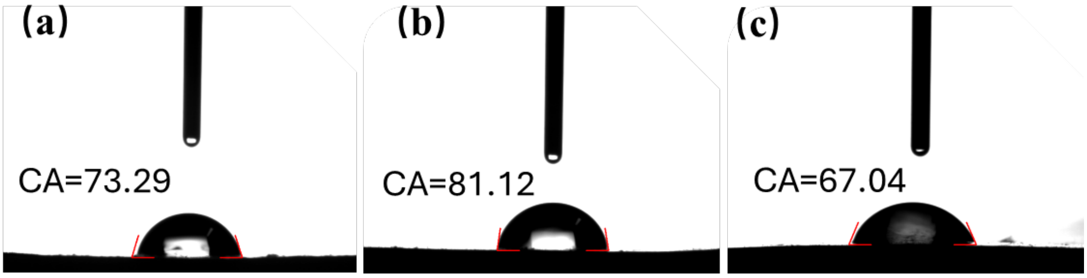


Figure S2. Contact angle of WPU (a), $SiO_2$/EGP/WPU (b) and $SiO_2$@PDA-G/WPU (c).
